# Evanescent wave in multiple slit diffraction and n-array antennas in metamaterial using Cesàro convergence

**DOI:** 10.1038/s41598-023-36894-8

**Published:** 2023-06-20

**Authors:** Yuganand Nellambakam, K. Haritha, K. V. S. Shiv Chaitanya

**Affiliations:** 1 Department of Physics, BITS Pilani, Hyderabad Campus, Jawahar Nagar, Shamirpet Mandal, Hyderabad, 500 078 India; 2 Present Address: Department of Science and Humanities, MLR Institute of Technology, Hyderabad, Telangana 500043 India; 3 Department of Physics, Govt Degree College, Alair, Yadadri Bhuvanagiri Dist, Telangana 508101 India

**Keywords:** Optics and photonics, Physics

## Abstract

In this paper, we study Multiple slit diffraction and n- array linear antennae in negative refractive index material. We show that the evanescent wave plays a vital role in the near-field term. The evanescent wave grows significantly, unlike in conventional materials, and satisfies a novel kind of convergence known as Cesàro convergence. We calculate the intensity of multiple slits and the antenna’s amplification factor (AF) in terms of the Riemann zeta function. We further demonstrate that the Riemann zeta function gives rise to additional nulls. We deduce that all the diffraction scenarios in which the traveling wave satisfies the geometric series in the medium of the positive refractive index will enhance the evanescent wave, which satisfies Cesàro convergence in the medium of the negative refractive index.

## Introduction

An evanescent wave is an oscillating electric and/or magnetic field that does not travel as an electromagnetic wave instead, it is spatially concentrated around the source. This is a near-field area phenomenon whose magnitude decays exponentially with increasing distance. It can be represented mathematically by a wave vector with one or more of its components having an imaginary value. In optics, the transmitted field in total internal reflection is an evanescent wave^1–9^, the evanescent-wave coupling is the technique of transmitting electromagnetic waves from one material to another^1–9^. This coupling is often achieved by arranging two or more electromagnetic devices, such as optical waveguides, so close to each other that the evanescent field created by one element does not decay significantly before reaching the other. If receiving waveguide supports modes of the proper frequency, the evanescent field generates propagating-wave modes, connecting (or coupling) the wave from one waveguide to the next^1–9^. In photonic and nanophotonic devices, evanescent wave coupling is frequently employed as waveguide sensors^1–9^. In electromagnetic field theory, evanescent-wave coupling and the near field interaction are basically the same^1–9^.

Pendry has shown how evanescent waves, which are effectively responsible for subwavelength resolution, may increase exponentially inside a well-built slab of double-negative (DNG) material, which is known as metamaterials, and how this effect can “compensate” for the fading exponential occurring outside the slab^10^. In a realizable negative refractive index lens, the periodicity of the negative refractive index medium limits the amplitude of the rising evanescent waves. It prevents them from increasing to unphysically enormous levels studied^11^. The study shows that the growing evanescent waves lead to the largest field amplitude at the second interface of the lens^12^. Pendry’s lens and related “growing exponential” and subwavelength imaging systems (see, e.g.,^13–17^). In a slab with negative permittivity or negative permeability, it was shown that forward and backward evanescent surface waves occur^18^. A medley of simulations of the full-wave and circuit demonstrating a grid of continual micro-strip transmission lines can forge evanescent waves in the lack of any lumped portions^19^. The dual-Transmission-line(TL) structure is an analytical formulation that depicts the negative refraction of propagating waves and the growth of evanescent waves within a negative refractive index lens constructed with a periodical L, C loaded TL network^20^. Anthony Grbic *et al.* have demonstrated a growing evanescent wave in a dual-TL structure through simulation^12^.

The metamaterial has two main features: one being backward waves and another the amplification of evanescent waves. The first feature triggers many unusual phenomena like negative refraction, the reversed Doppler effect, and the reversed Cherenkov radiation, etc. The second feature can find essential applications in the subwavelength focusing, super-resolution imaging, and high-capacity storage. The amplification of evanescent waves has been shown to be able to enhance the interaction between wave and matter and then increase the sensitivity of sensors. The experimental verification of evanescent wave amplification (EWA) has been studied in^21,22^. In order to observe the exponentially increasing-decreasing field distribution in EWA, a series-shunt capacitor (CC) and series-shunt inductor (LL) to simulate artificial magnetic plasma and electric plasma has been proposed^23^.

In this paper, we show that applying cesàro convergence enhances the amplification of evanescent waves in n-array negative refractive material antennas. Two of the authors, in their previous work, have shown that the linear dielectrics and magnetic materials in the matter^24^ and the restoration of the evanescent wave in a perfect lens^25^ obey a unique convergence called Cesàro convergence.

### Multiple slit diffraction

The underlying physical principle of image formation is diffraction. In a perfect lens, an evanescent wave plays an important role in image formation. A natural question that emerges is whether an evanescent wave enhances negative refractive materials during diffraction. To this, we study multiple slit diffraction. Consider the geometry for diffraction with multiple slits. For *N* slits, the net contribution to the field $E_P$^26,27^ is given by

1 $$\begin{aligned} E_P=\sum _{n=0}^{N-1}\int _{na-D/2}^{na+D/2}\;\;\;\;\; dE_p. \end{aligned}$$

Using the Fraunhofer diffraction approximations, the Eq. (1) can be written as

2 $$\begin{aligned} E_P=\dfrac{E_L}{R}\sum _{n=0}^{N-1}\;\;\;\;\;sin(\omega t-kR+xksin\theta )\;\;dx. \end{aligned}$$

Concentrating on the x-dependent component of this interaction, we have

3 $$\begin{aligned} \sum _{n=0}^{N-1}\Big [\frac{e^{ikx\;sin\theta }}{ik\;sin\theta }\Big ]_{na-D/2}^{na+D/2}= & {} \sum _{n=0}^{N-1}e^{ikna\;sin\theta }D\frac{sin(k(D/2)sin\theta )}{(k(D/2)sin\theta )}\nonumber \\= & {} \sum _{n=0}^{N-1}e^{in2\alpha }D\frac{sin\beta }{\beta } \end{aligned}$$

Defining

4 $$\begin{aligned} \alpha =\dfrac{ka}{2}sin\theta \;\;\;\;\;\;\beta = k(D/2)sin\theta \end{aligned}$$

Then the intensity takes the form(see Supplementary Information 1)

5 $$\begin{aligned} I(\theta )=I(0)\Big (\dfrac{sin\;N\alpha }{N\; sin\;\alpha }\Big )^2\Big (\dfrac{sin\;\beta }{\beta }\Big )^2 \end{aligned}$$

### Multiple slit diffraction in negative refractive index

From Multiple slit Diffraction geometry, adding $\phi$ to the phase element of the x-dependent component interaction (3), we have

6 $$\begin{aligned} \sum _{n=0}^{N-1}\Big [\frac{e^{i\;k\;x\;sin\theta +\phi }}{i\;k\;sin\theta +\phi }\Big ]_{na-D/2}^{na+D/2}= & {} \sum _{n=0}^{N-1}e^{i\;k\;n\;a\;sin\theta +\phi }D\frac{sin(k(D/2)sin\theta +\phi )}{(k(D/2)sin\theta +\phi )} \end{aligned}$$

7 $$\begin{aligned}= & {} \sum _{n=0}^{N-1}e^{i\;n\;2\;\alpha +\phi }D\frac{sin\beta }{\beta } \end{aligned}$$

where we defined $\alpha = \frac{ka}{2}sin\theta$ and $\beta = (k(D/2)sin\theta +\phi )$.

8 $$\begin{aligned} S_N = \sum _{n=0}^{N-1}e^{i\;n\;2\;\alpha +\phi } \end{aligned}$$

substituting $\alpha$ value in the Eq. (8) and finally (see the calculations in Supplementary Information 2), we get

9 $$\begin{aligned} S_N= & {} \sum _{s=0}^{N-1}(-1)^s \;e^{s\;n_{im}\kappa \;sin\theta +\phi ^{\prime }}\;\cdot \; \sum _{s=0}^{N-1}e^{isn_r\;\kappa \;sin\theta +\phi ^{\prime }}\nonumber \\ \end{aligned}$$

Let $$\Psi =\kappa \;sin\theta +\phi ^{\prime }$$

10 $$\begin{aligned} S_N = \sum _{s=0}^{N-1}(-1)^s \;e^{s\;n_{im}\Psi }\;\cdot \;\sum _{s=0}^{N-1}e^{isn_r\;\Psi } \end{aligned}$$

From the Eq. (10), it is clear that an evanescent wave does not decay but grows exponentially. Therefore we study the convergence of the evanescent wave using Cesàro convergence.

### Linear antenna arrays

The other application, which is directly related to diffraction, is the antenna. One example that resembles multiple-slit diffraction is n-linear antenna arrays. We study its effect in the presence of negative refractive index materials or metamaterial.

The metamaterial antennas, their production, application, and research are gaining prominence rapidly. Several strategies have been explored during the past decade to enhance antenna performance. The employment of metamaterials in antenna design is one such method. The metamaterial form antenna optimizes antenna performance by employing the innovative functionality of the metamaterials. The peculiar properties of these materials enable the creation of high-performance antennas, filters, and microwave devices that can not be attained with traditional antennas. Negative refractive indices, chiral left-handed materials, single negative metamaterials, electromagnetic bandgap left-handed materials, double positive medium, bi-isotropic, and anisotropic backward-wave media are some of the electromagnetic metamaterial compositions available for use in the antenna field^28,29^

Leaky-wave antennas (LWAs)^30^ and small resonator-type antennas(SRAs) are the two most common types of metamaterial-based antennas. Metamaterial-associated small antennas are suggested to offer a mechanism for modifying the dispersion relationship or conditions at the near-field boundary, which might lead to antenna size reduction while retaining decent radiation efficacy. Metamaterial-associated antennas facilitate small antennas to circumvent the confined efficiency and bandwidth restriction. However, this technique is still far from being robust. LWA’s executed by the composite right/left-handed (CRLH) transmission line (TL) left-handed materials stimulate the backward to forward beam scanning, incorporating the broadside radiation, which is tough to accomplish with traditional LWAs^30–34^.

## Two-element array

The most basic geometry of an array is a linear array, where every component is oriented along a straight line. The minimal span linear array is the two-element array. Consider electric fields $E_1$ and $E_2$ in the distant locality of the array elements^35–38^

11 $$\begin{aligned} {\textbf {E}}_1=M_1E_{n1}(\theta _1, \phi _1)\frac{e^{-j\left( kr_1-\frac{\beta }{2}\right) }}{r_1}\hat{\pmb {\rho }}_1,\;\; {\textbf {E}}_2=M_2E_{n2}(\theta _2, \phi _2)\frac{e^{-j\left( kr_2+\frac{\beta }{2}\right) }}{r_2}\hat{\pmb {\rho }}_2, \end{aligned}$$

here $M_1$ and $M_2$, -size of field (magnitudes), $E_{n1}$ and $E_{n2}$-uniform (normalized) field patterns, $r_1$ and $r_2$-distances from P, $\beta$-phase difference(between 2array elements), and $\hat{\pmb {\rho }}_1$ and $\hat{\pmb {\rho }}_2$ are far-zone E fields polarization vectors. Assuming the array elements are similar, have identical polarizations $\hat{\pmb {\rho }}_1=\hat{\pmb {\rho }}_2=\hat{\pmb {\rho }}$, and have excitations of the same amplitude. By taking $E_{n_{1}}(\theta , \phi )=E_{n_{2}}(\theta , \phi )=E_{n}(\theta , \phi )$, and $M_1=M_2=M$, the electric field is given by

12 $$\begin{aligned} {\textbf {E}}={\textbf {E}}_1+{\textbf {E}}_2=\underbrace{\hat{\pmb {\rho }}M \dfrac{e^{-jkr}}{r}E_n(\theta , \phi ) }_{\begin{array}{c} \text {Entire field} \end{array}}\times \underbrace{2cos\left( \dfrac{kd\;cos\theta +\beta }{2}\right) }_{\begin{array}{c} \text {Array factor} \end{array}} \end{aligned}$$

The array’s full field is equivalent to the product of the field created by a single element at the origin (element factor) and the array factor (AF):

13 $$\begin{aligned} AF_n=cos\left( \dfrac{kd\;cos\theta +\beta }{2}\right) \end{aligned}$$

In the Eq. (13), we use normalized array factor, $E_n(\theta ,\phi )$, and single element normalized field pattern.

### n-Array

A uniform array is a group of related objects that possess the same magnitudes and progressive phases. It is possible to determine the AF of the uniform array by seeing each element as a single point (isotropic) source. The AF of an N-element linear array of isotropic sources is^35–38^

14 $$\begin{aligned} AF= 1+e^{j(kd\;cos\theta +\beta )}+e^{j2(kd\;cos\theta +\beta )}+\cdots +e^{j(N-1)(kd\;cos\theta +\beta )}. \end{aligned}$$

Eq. (14) can be re-written as

15 $$\begin{aligned} AF = \sum ^N_{n=1}e^{j(n-1)\Psi } \end{aligned}$$

where $\Psi =kd\;cos\theta +\beta$. The normalized AF and neglecting phase effect result in

16 $$\begin{aligned} AF = \dfrac{sin(N\Psi /2)}{sin(\Psi /2)}. \end{aligned}$$

For small values of $\Psi$ Eq. (16) leads to

17 $$\begin{aligned} AF= \dfrac{sin(N\Psi /2)}{\Psi /2}. \end{aligned}$$

We need the maximum of the AF to normalize Eq. (16) or (17). When $\psi =0$, the maximum value of AF arises. The result AF = N, ignoring the phase factor, the normalized AF, is thus obtained

18 $$\begin{aligned} AF= \dfrac{1}{N}\left[ \dfrac{sin(N\Psi /2)}{\Psi /2}\right] . \end{aligned}$$

### Cesàro convergence

We present a brief description of Cesàro convergence. Consider the series

19 $$\begin{aligned} Q=1-1+1-1+1 \cdots =\sum _{n=0}^{+\infty }Q_j=\sum _{n=0}^{+\infty }(-1)^n. \end{aligned}$$

This series (19) is referred to as Grandi’s series in the literature. Grandi’s series doesn’t quite meet the standard geometric convergence; that is, the sum to infinity $\sum _{0}^{\infty }x^n=\frac{1}{1-x}$ is not specified. Typically, for a geometric series to converge, *x* should fall between $-1$ and 1, i.e.,$-1<x<1$; in this instance, − 1 and 1 are also excluded. In Grandi’s series, the value of *x* is $x=-1$. It is interesting to note that Ramanujan^40^ has used the value of $x=-1$ in the geometric series sum to infinity $\sum _{0}^{\infty }x^n=\frac{1}{1-x}$ and obtained the value of $\sum _{0}^{\infty }x^n=1/2$. The value of 1/2 as the sum to infinite series in Eq. (19) is justified if we assume that the Grandi’s series obeys Cesàro convergence. Here is a brief description of Cesàro convergence: For a geometric series to converge, the sequence of partial sums should converge to a real number. The sequence of partial sums for Grandi’s series gives

20 $$\begin{aligned}&P_0=Q_0=1,;\;\;\;\;P_1=Q_0+Q_1=0,;\;\;\;\;P_2=Q_0+Q_1+Q_2=1, ;\nonumber \\ {}&P_3=Q_0+Q_1+Q_2+Q_3=0,..... \end{aligned}$$

It is clear from Eq. (20) that the sequence of partial sums does not converge to a real number. But, the sum to infinity of geometric series gives $\sum _{0}^{\infty }(-1)^n=\frac{1}{1-(-1)}=\frac{1}{2}$, a real number. The RHS converges, and LHS diverges, hence for consistency, we consider the averages of partial sums, that is,

21 $$\begin{aligned} \frac{P_0}{1}=1,;\;\;\;\ \frac{P_0+P_1}{2}=\frac{1}{2},;\;\;\;\ \frac{P_0+P_1+P_2}{3}=\frac{2}{3},;\;\;\;\ \frac{P_0+P_1+P_2+P_3}{4}=\frac{2}{4} \end{aligned}$$

and so on. Sequence of the average of partial sums gives

22 $$\begin{aligned} P_n = \frac{1}{n}\sum _{k=1}^{n}Q_n= \frac{1}{1},\frac{1}{2},\frac{2}{3},\frac{2}{4},\frac{3}{5}, \frac{3}{6},\frac{4}{7},\frac{4}{8},\frac{5}{9},\frac{5}{10},... \end{aligned}$$

The Eq. (22) is re-casted as

23 $$\begin{aligned} P_n={\left\{ \begin{array}{ll} \frac{1}{2}, &{}for \;\;n \;\;odd\\ \frac{1}{2}+\frac{1}{2n+2}, &{}for\;\; n \;\;even \end{array}\right. } \end{aligned}$$

as *n* goes to infinity, it converges to $\frac{1}{2}$. From the Eq. (22), as the average of partial sums converges to a real number, it’s a Cesàro Convergence. A series $\sum _{j=0}^{n} a_j$ is Cesàro summable if this satisfies the following theorem:

#### Theorem 1

Suppose $\sum _{j=0}^{n} a_j$ is a series that converges and it has sum, say L. Consequently $\sum _{j=0}^{n} a_j$ is Cesàro summable to L.

24 $$\begin{aligned} \lim _{n\rightarrow \infty }s_n = L \in \mathbb {R}\;\;\;\;\Rightarrow \;\;\;\lim _{n\rightarrow \infty }\sigma _{n} = L \in \mathbb {R}. \end{aligned}$$

The proof is given in^41^. The properties of Cesàro sums: If $\sum _n a_n$ = A and $\sum _n b_n$ = B are convergent series, then

- Sum-Difference Rule: $\sum _n(a_n\pm b_n)$ = $\sum _n a_n \pm \sum _n b_n$ = A ± B.
- Constant Multiple Rule : $\sum _n c\; a_n$ = c $\sum _n a_n$ = cA for any real number c.
- The product of $AB=\sum _n a_n\sum _n b_n$ also as Cesàro sums.

Applying theorem 1 we get

25 $$\begin{aligned} \lim _{n\rightarrow \infty }\; P_{2n} = \lim _{n\rightarrow \infty }P_{2n+1} = \frac{1}{2}. \end{aligned}$$

For more details, readers may refer to^24^. A surprising result on summability asserts that statistical convergence and high Cesàro convergence are inextricably linked^42,43^. Within the strong p-Cesàro convergence framework, a new version of the Orlicz-Pettis theorem is proposed^44^.

## Application of Cesàro convergence to linear antenna arrays

An antenna array is a series of equivalent miniature antennas that collectively generate a signal comparable to that of a giant antenna. We will now examine the evanescent wave in an n-array metamaterial antenna using Cesàro convergence. Consider the Eq. (17)

26 $$\begin{aligned} AF = \sum ^N_{q=1}e^{i(q-1)(kd\;cos\theta +\beta )} \end{aligned}$$

By defining the refractive index in complex form and re-writing the Eq. (26), we get

27 $$\begin{aligned} AF = \sum ^N_{q=1}e^{i(q-1)\big (\dfrac{\omega }{c}(n_r-in_{im})d \;cos\theta +\beta \big )}\;\;\;\;\big (\because \tilde{k}=\dfrac{\omega \tilde{n}}{c}\big ) \end{aligned}$$

The Eq. (27) can be rewritten into the following form if ’i’ is included inside the brackets:

28 $$\begin{aligned} AF = \sum ^N_{q=1}e^{(q-1)\big (\dfrac{\omega }{c}(n_{im}+in_r)d\;cos\theta +\beta \big )} \end{aligned}$$

Let $$\dfrac{\omega }{c}d =\kappa$$ & $$(q-1) =l$$

29 $$\begin{aligned} AF = \sum ^{N-1}_{l=0}e^{l\;\big ((n_{im}+in_r)\kappa \;cos\theta +\beta \big )} \end{aligned}$$

Defining $$\beta =\beta +\pi$$

30 $$\begin{aligned} AF = \sum ^{N-1}_{l=0}e^{l\;\big ((n_{im}+in_r)\kappa \;cos\theta +(\beta +\pi ) \big )} \end{aligned}$$

Rearranging,

31 $$\begin{aligned} AF = \sum ^{N-1}_{l=0}(-1)^{l}e^{l\;\big ((n_{im}+in_r)\kappa \;cos\theta +\beta \big )} \end{aligned}$$

Let $$\psi =(\kappa \;cos\theta +\beta )$$

32 $$\begin{aligned} AF = \sum ^{N-1}_{l=0}(-1)^{l}e^{l\;(n_{im})\;\psi }\;\;\cdot \sum ^{N-1}_{s=0}e^{s(in_r)\;\psi } \end{aligned}$$

Euler obtained Riemann Zeta ($\zeta$) function using the following sequence

33 $$\begin{aligned} e^{-y}-e^{-2y}+e^{-3y}-e^{-4y}+\cdots =\frac{1}{e^y+1}, \end{aligned}$$

which converges for any y > 0. For $y=0$, the Eq. (33) simplifies to Grandi’s series. Using $y=-(n_{im}\;\psi )$ we can write Eq. (32) as

34 $$\begin{aligned} AF = \sum ^{N-1}_{l=0}(-1)^{l}e^{-yl}\;\;\cdot \sum ^{N-1}_{s=0}e^{i(sn_r)\;\psi } \end{aligned}$$

When we differentiate the Eq. (33) $(n-1)$ times, we obtain

35 $$\begin{aligned} 1^ne^{-y}-2^{n}e^{-2y}+3^{n}e^{-3y}-4^ne^{-4y}+\cdots =(-1)^n\frac{d^n}{dy^n}\left( \frac{1}{e^y+1}\right) , \end{aligned}$$

which, for every y> 0, converges once more. Then by extending the function $1/(e^y+1)$ around $y=0$ using Taylor series, we obtain

36 $$\begin{aligned} \frac{1}{e^y+1} = \sum _{k=0}^{\infty } a_k y^{k} \end{aligned}$$

Euler, by taking *k* to be complex in Eq. (36), obtained the following functional version of Riemann Zeta function^45,46^

37 $$\begin{aligned} \zeta (1-l)=2(2\pi )^{-l}cos\left( \frac{l\pi }{2}\right) \Gamma (l)\zeta (l), \end{aligned}$$

From (35), (36) and (37) the first term in the Eq. (34) becomes

38 $$\begin{aligned} \sum ^{l-1}_{k=0}(-1)^{k}e^{-yk}=2(2\pi )^{-l}cos\left( \frac{l\pi }{2}\right) \Gamma (l)\zeta (l) \end{aligned}$$

From (18) and (16) the second term in the Eq. (34) becomes

39 $$\begin{aligned} \sum ^{N-1}_{s=0}e^{i(sn_r)\;\psi }=\dfrac{sin(N\Psi /2)}{Nsin(\Psi /2)} \end{aligned}$$

From Eq. (38) and (39) Eq. (34) can be written as

40 $$\begin{aligned} AF=2(2\pi )^{-l}cos\left( \frac{l\pi }{2}\right) \Gamma (l)\zeta (l)\cdot \;\;\dfrac{sin(N\Psi /2)}{Nsin(\Psi /2)} \end{aligned}$$

### Application of Cesàro convergence to multiple slit diffraction

Consider the Eq. (10), let $y=-n_{im}\Psi$, then the Eq. (10) can written as

41 $$\begin{aligned} S_N=\sum _{s=0}^{N-1}(-1)^s \;e^{-ys}\;\cdot \;\sum _{s=0}^{N-1}e^{i\;s\;n_r\;\Psi } \end{aligned}$$

the Eq. (41) is exactly identical to Eq. (32). Applying Cesàro convergence we get

42 $$\begin{aligned} S_n=2(2\pi )^{-s}cos\left( \frac{s\pi }{2}\right) \Gamma (s)\zeta (s)\; \cdot \;\dfrac{sin(N\alpha )}{Nsin(\alpha )}\dfrac{sin(\beta )}{(\beta )} \end{aligned}$$

The intensity takes the form

43 $$\begin{aligned} I=I_0\left[ 2(2\pi )^{-s}cos\left( \frac{s\pi }{2}\right) \Gamma (s) \zeta (s)\right] ^2\;\cdot \;\left[ \dfrac{sin(N\alpha )}{Nsin(\alpha )}\right] ^2\left[ \dfrac{sin(\beta )}{(\beta )}\right] ^2 \end{aligned}$$

## Results and discussion

We have investigated two scenarios: multiple slit and n-array linear antennas in a negative refractive index where the evanescent wave enhances and will not die. In the first case, we computed the AF(Amplification Factor) in Eq. (40), and in the second case, we calculated the intensity in Eq. (43). In the case of linear antenna, as it is clear from Eq. (27) when it is placed in negative refraction, the evanescent wave does not dampen. Similarly, in the case of multiple slit diffraction, it is clear from Eq. (41) evanescent wave does not dampen and will grow indefinitely. By applying Cesàro convergence, we demonstrate that the evanescent wave does indeed exhibit convergence as indicated by Eq. (40) and Eq. (43). We have analyzed both scenarios and plotted them below.

**Figure 1:**
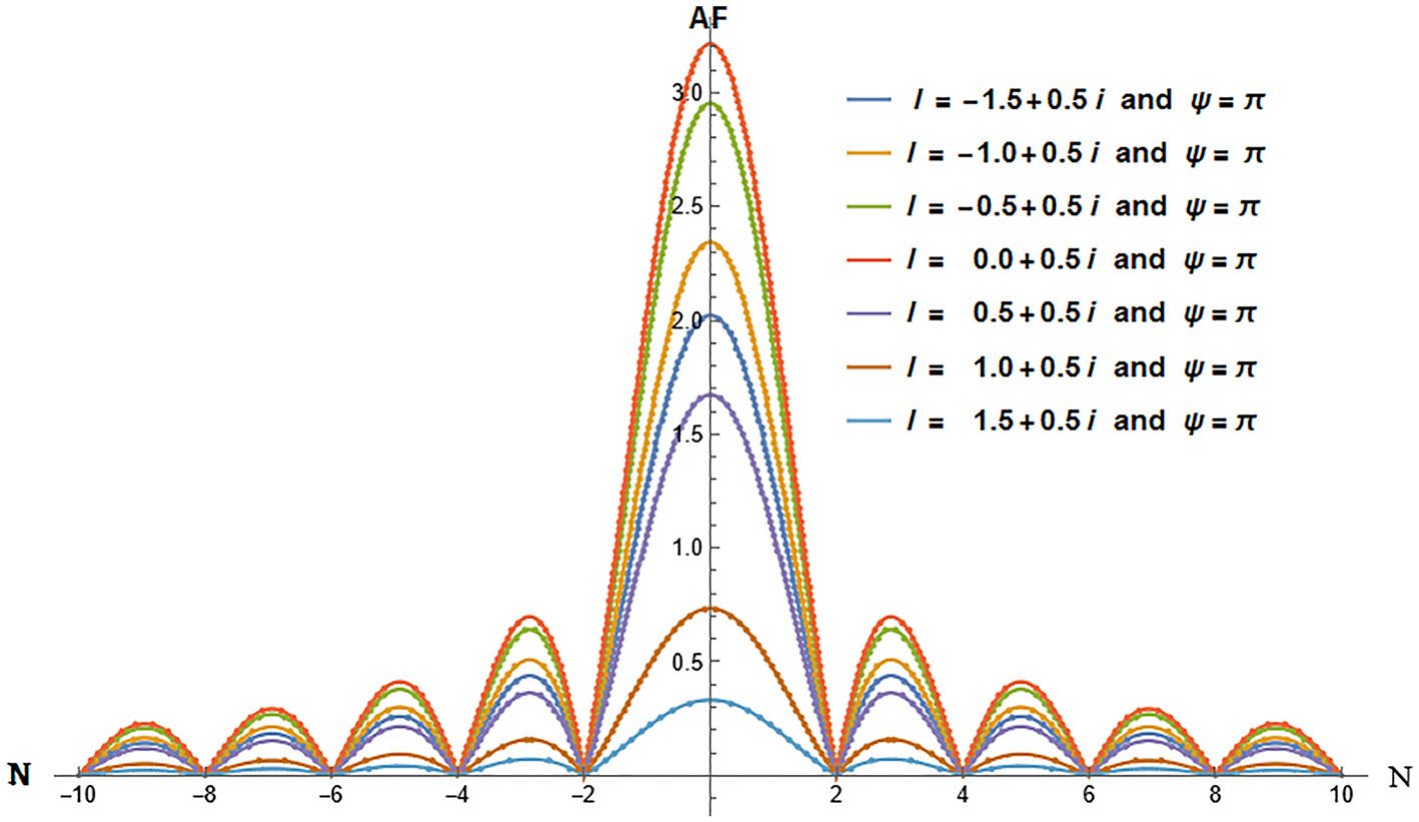
Amplification Factor versus N. We can observe the growth of an evanescent wave when we vary *l*, which is similar to the diffraction pattern.

**Figure 2:**
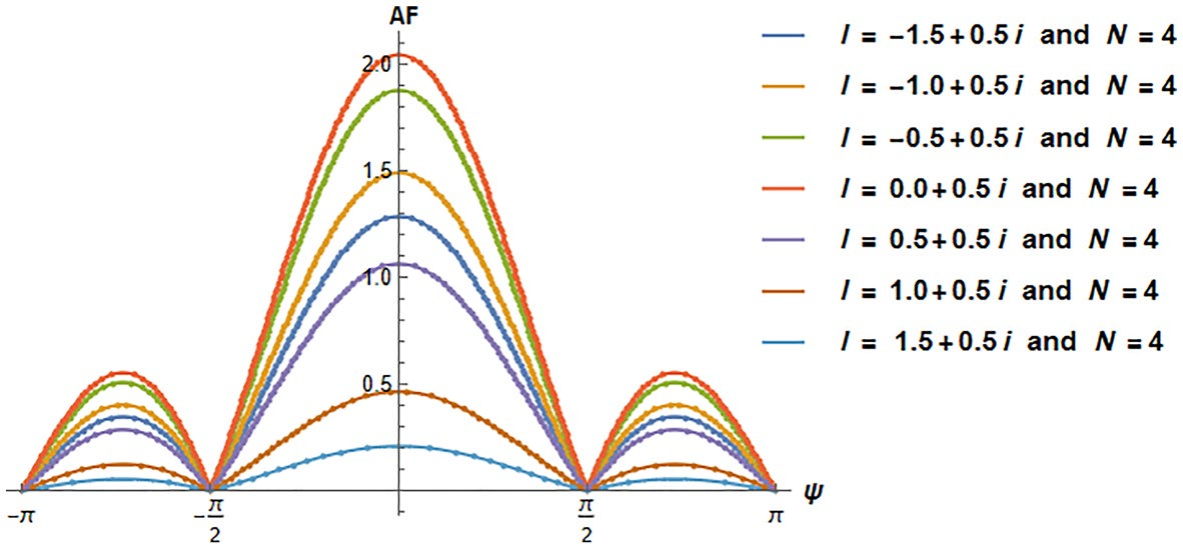
Amplification Factor versus $\Psi$. When we vary $\Psi$, the evanescent wave enhances in one full wavelength.

**Figure 3:**
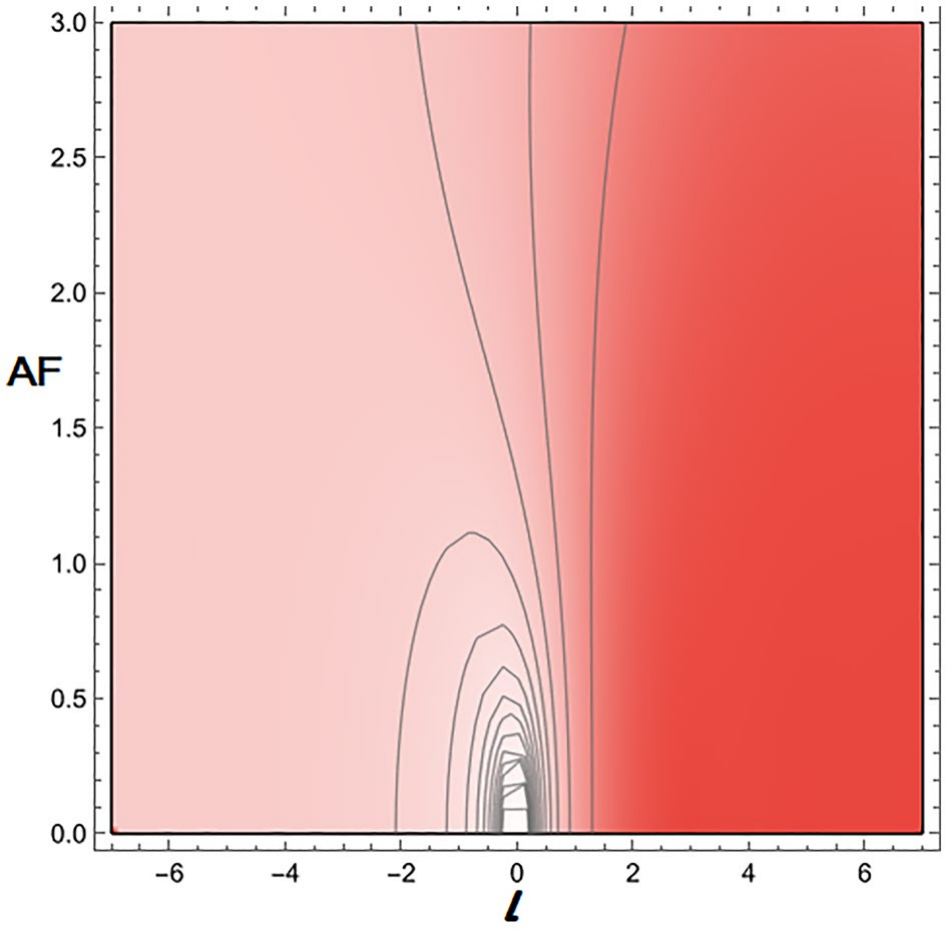
Amplification Factor versus *l*. The varying parameter *l* related to the Reimann zeta function helps to enhance the evanescent wave and is respo-nsible for more nulls.

From Fig. 1, it is evident that the evanescent wave is amplified in the negative refractive medium, where we recover the diffraction pattern as we vary N(N=-10 to 10) for a fixed value of $\Psi$($\Psi =\pi$) and different values of *l*(*l* = − 1.5 + 0.5i, − 1 + 0.5i, − 0.5 + 0.5i, 0 + 0.5i, 0.5 + 0.5i, 1.0 + 0.5i, 1.5 + 0.5i) in Eq. (40). In Fig. 2, we observe that for the fixed value of *N* (N = 4) and *l*(*l* = − 1.5 + 0.5i, − 1 + 0.5i, − 0.5 + 0.5i, 0 + 0.5i, 0.5 + 0.5i, 1.0 + 0.5i, 1.5 + 0.5i), the pattern repeats after every $2\pi$ by varying $\Psi$ ($\Psi$=$-\pi$ to $\pi$) in Eq. (40). In Fig. 3, we vary *l*(*l* = − 7 to 7 + 3i) by fixing N(N=2) and $\Psi$($\Psi$ = $\pi$). It should be clear to the readers that the evanescent wave amplifies the AF. Even it also vanishes on the imaginary line, giving rise to new nulls absent in the natural materials. The nulls occur when AF is zero, which gives the condition^35–38^. From Eq. (40), we get additional nulls from the Riemann zeta function, which vanishes at $l=2$ as shown in Fig. 1 and also at − 6, − 4, − 2, 0, 2, 4, and 6.

**Figure 4:**
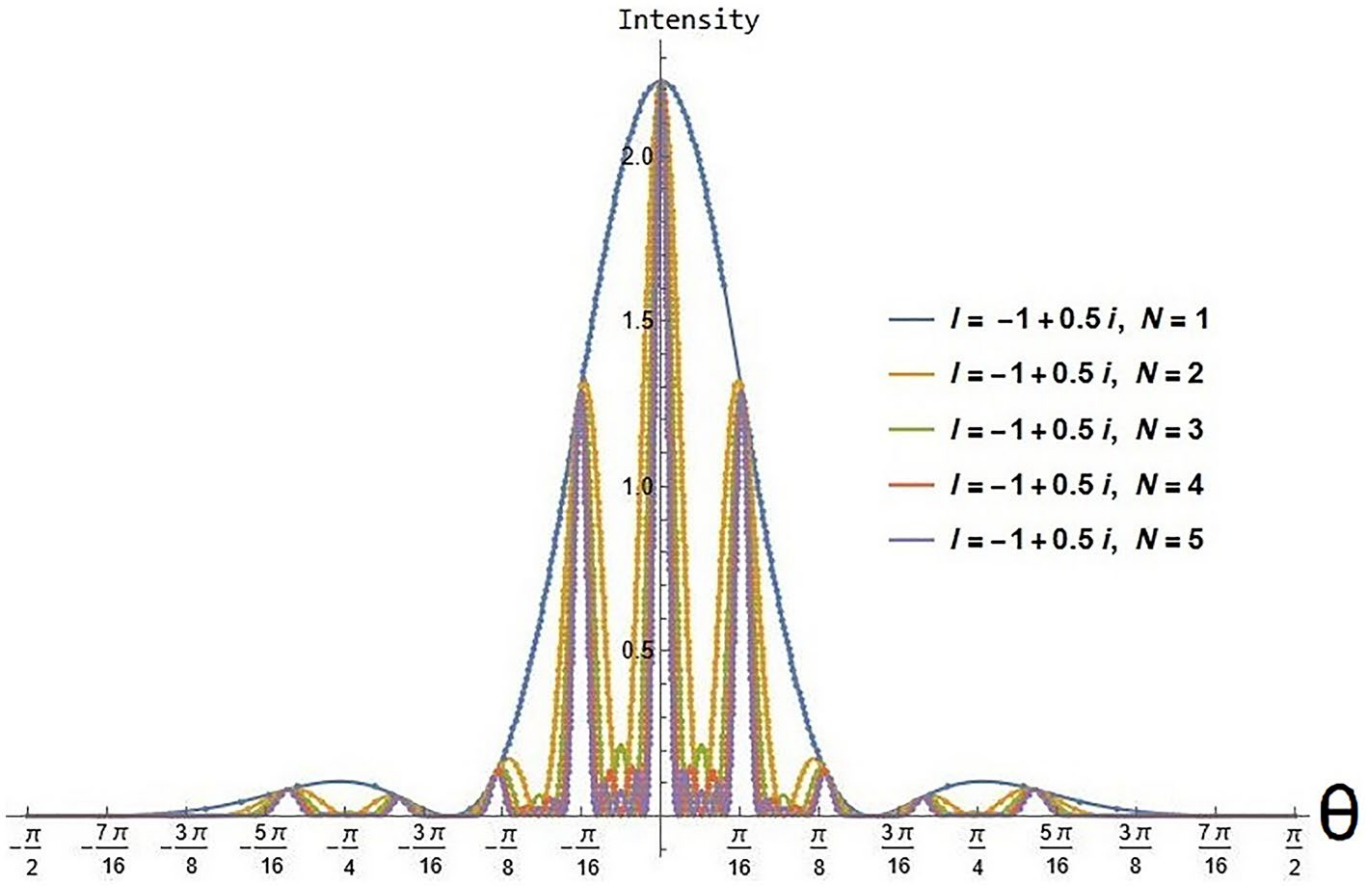
Intensity versus Angle of diffraction($\theta$). By varying $\theta$ with different values of *N* and constant *l* value gives the diffraction pattern which replicates the original one with enhancing parameter.

From Fig. 4, intensity versus $\theta$($\theta$ = − $\pi /2$ to $\pi /2$) plot, the evanescent wave clearly enhances the electric field’s intensity for fixed values of *l*(*l* = − 1 + 0.5i) and different values of N (N = 1 to 5) in Eq. (43) (where $\phi$ is taken 0). Readers should note that the diffraction condition does not change for the traveling wave. But we get new conditions for the minimum as the intensity equation in terms of the Riemann zeta function gives zero for particular values of *l*, i.e., $l=-2s$, where *s* is an integer, the Riemann zeta function also has a unique zero on the critical line, also known as the Riemann hypothesis. The demonstration of these zero is the restatement of the Riemann hypothesis.

The enhancement of evanescent waves is a well-studied problem in the case of superlens or perfect lenses. t’Hooft commented^39^ on Pendry’s perfect lens^10^, where he addressed the geometric series of a convergence problem. To this, Pendry still needs to give a satisfactory reply. The authors of this paper have shown that one can recover Pendry’s results using Cesàro Convergence. Readers should note that historically convergence has always led to physical reality. A well-known example is the Zero paradox, which was solved by calculus. Our approach to solving the enhancement of evanescent waves glorifies metamaterials. The electric field of Several coherent oscillators^26^ satisfies geometric series, is given by

44 $$\begin{aligned} \tilde{E}=E_0(r)e^{-i\omega t}e^{[ikr_1+(N-1)\delta /2]}\left( \frac{sin N\delta /2}{sin\delta /2}\right) . \end{aligned}$$

It should be clear to the readers when Several coherent oscillators placed in a negative refractive index medium satisfy Cesàro convergence.

## Conclusion

In this paper, we studied Multiple slit diffraction and n- array linear antennae in negative refractive index material. We have shown that the evanescent wave plays a vital role in the near-field term. The evanescent wave grows significantly, unlike in conventional materials, and satisfies a novel kind of convergence known as Cesàro convergence. We calculated the intensity of multiple slits and the antenna’s amplification factor (AF) in terms of the Riemann zeta function. We further demonstrated that the Riemann zeta function gives rise to additional nulls for *l* being real and has zeros on the critical line, known as the Riemann hypothesis. In the case of diffraction, the diffraction condition does not change for the traveling wave. But we get new conditions for the minimum as the intensity equation in terms of the Riemann zeta function gives zero for particular values of *l*, i.e., $l=-2s$, where *s* is an integer, and the Riemann zeta function also has a unique zero on the critical line when the real part is 1/2, also known as the Riemann hypothesis. The demonstration of these zero is the restatement of the Riemann hypothesis. We have also shown that the electric field of Several coherent oscillators also satisfies Cesàro convergence in the negative refractive medium. Therefore, we conclude all the diffraction scenarios in which the traveling wave satisfies the geometric series in the medium of the positive refractive index will enhance the evanescent wave, which satisfies Cesàro convergence in the medium of the negative refractive index.

## Supplementary Information

Supplementary Information.

## Data Availability

The datasets used and analyzed during the current study are available from the corresponding author upon reasonable request.
