# Supplementary material for: Evanescent wave in multiple slit diffraction and n-array antennas in metamaterial using Cesàro convergence
Source: Sci Rep. 2023 Jun 20;13:9981. doi: 10.1038/s41598-023-36894-8 (PMC10282094; doi:10.1038/s41598-023-36894-8)
Supplement: Supplementary file 1 — Supplementary Information. [file 41598_2023_36894_MOESM1_ESM.pdf]

SUPPLEMENTARY INFORMATION

Evanescent wave in Multiple slit diffraction and n-array Antennas in Metamaterial using Cesàro convergence

YUGANAND NELLAMBAKAM[1], K.HARITHA[2], and K.V.S SHIV CHAITANYA[1*]

[1]Department of Physics,
BITS Pilani, Hyderabad Campus,
Jawahar Nagar, Shamirpet Mandal,
Hyderabad, India 500 078.
[2]Department of Physics,
Govt Degree College, Alair,
Yadadri Bhuvanagiri dist,
Telangana, India 508101.
[*]chaitanya@hyderabad.bits-pilani.ac.in

SUPPLEMENTARY INFORMATION 1

Multiple slit diffraction

Diffraction is the basic physical principle of image formation. An evanescent wave is crucial to developing an image in a perfect lens. Whether an evanescent wave increases negatively refractive materials during diffraction is a natural question that arises. We research multiple slit diffraction in relation to this. Consider the diffraction geometry for numerous slits. The net contribution to the field for N slits E_P[1,2] is given by

$$E_P = \sum_{n=0}^{N-1} \int_{na-D/2}^{na+D/2} dE_p. \tag{1}$$

Using the Fraunhofer diffraction approximations, the eq(1) can be written as

$$E_P = \frac{E_L}{R} \sum_{n=0}^{N-1} sin(\omega t - kR + xksin\theta)\ dx. \tag{2}$$

Concentrating on the x-dependent component of this interaction, we have

$$\begin{aligned} \sum_{n=0}^{N-1} \left[\frac{e^{ikx\,sin\theta}}{ik\,sin\theta}\right]_{na-D/2}^{na+D/2} &= \sum_{n=0}^{N-1} e^{ikna\,sin\theta} D \frac{sin(k(D/2)sin\theta)}{(k(D/2)sin\theta)} \\ &= \sum_{n=0}^{N-1} e^{in2\alpha} D \frac{sin\beta}{\beta} \end{aligned} \tag{3}$$

Defining

$$\alpha = \frac{ka}{2} sin\theta \qquad \beta = k(D/2)sin\theta \tag{4}$$

In eq (3), the remaining factor is a geometric progression with a common factor $e^{i2\alpha}$.

$$S_N = \sum_{n=0}^{N-1} e^{i2n\alpha} \tag{5}$$

Multiplying the eq (5) by $e^{i2\alpha}$, we get

$$S_N e^{i2\alpha} = \sum_{n=1}^{N} e^{i2n\alpha} \tag{6}$$

Subtracting eq (6) from eq (5), we get

$$S_N \left(1 - e^{i2\alpha}\right) = 1 - e^{i2N\alpha} \tag{7}$$

Therefore for S_N

$$\begin{aligned} S_N &= \frac{1 - e^{i2N\alpha}}{1 - e^{i2\alpha}} = \frac{e^{iN\alpha}\left(e^{-iN\alpha} - e^{iN\alpha}\right)}{e^{i\alpha}\left(e^{-i\alpha} - e^{i\alpha}\right)} \\ &= e^{i(N-1)\alpha} \frac{sinN\alpha}{sin\alpha}. \end{aligned} \tag{8}$$

Taking square modulus to the eq (8) drop down the phase factor $e^{i(N-1)\alpha}$ since

$$\lim_{\alpha \to 0} \frac{sinN\alpha}{sin\alpha} = N, \tag{9}$$

It is worthwhile to include a normalizing factor of 1/N in this ratio. Then the intensity takes the form

$$I(\theta) = I(0) \left(\frac{sin\ N\alpha}{N\ sin\ \alpha}\right)^2 \left(\frac{sin\ \beta}{\beta}\right)^2 \tag{10}$$

SUPPLEMENTARY INFORMATION 2

Multiple slit diffraction in negative refractive index

From Multiple slit Diffraction geometry, adding ϕ to the phase element of the x-dependent component interaction(3), we have

$$\begin{aligned} \sum_{n=0}^{N-1} \left[\frac{e^{i\,k\,x\,sin\theta + \phi}}{i\,k\,sin\theta + \phi}\right]_{na-D/2}^{na+D/2} &= \sum_{n=0}^{N-1} e^{i\,k\,n\,a\,sin\theta + \phi} D \frac{sin(k(D/2)sin\theta + \phi)}{(k(D/2)sin\theta + \phi)} && (11) \\ &= \sum_{n=0}^{N-1} e^{i\,n\,2\,\alpha + \phi} D \frac{sin\beta}{\beta} && (12) \end{aligned}$$

Where we defined $\alpha = \frac{ka}{2} sin\theta$ and $\beta = (k(D/2)sin\theta + \phi)$.

$$S_N = \sum_{n=0}^{N-1} e^{i\,n\,2\,\alpha + \phi} \tag{13}$$

substituting α value in the eq (13) we get

$$\begin{aligned}
S_N &= \sum_{s=0}^{N-1} e^{i\,s\,ka\,sin\theta+\phi}\left[\because \alpha = \frac{ka}{2} sin\theta\right] \\
&= \sum_{s=0}^{N-1} e^{i\,s\,\frac{\omega a}{c}\,n\,sin\theta+\phi}\left[\because k = \frac{\omega\, n}{c}\right] \\
&= \sum_{s=0}^{N-1} e^{i\,s\,\kappa\,n\,sin\theta+\phi}\left[\because \kappa = \frac{\omega\, a}{c}\right] \\
&= \sum_{s=0}^{N-1} e^{i\,s\,\kappa\,(n_r - in_{im})\,sin\theta+\phi}\left[\because n = n_r - in_{im}\right] \\
&= \sum_{s=0}^{N-1} e^{s(n_{im}+in_r)\,\kappa\,sin\theta+\phi} \\
&= \sum_{s=0}^{N-1} e^{s(n_{im}+in_r)\,\kappa\,sin\theta+\phi} \\
&= \sum_{s=0}^{N-1} (-1)^s\ e^{s(n_{im}+in_r)\,\kappa\,sin\theta+\phi'}\left[\because \phi = \phi' + \pi\right] \\
&= \sum_{s=0}^{N-1} (-1)^s\ e^{s\,n_{im}\kappa\,sin\theta+\phi'} \cdot \sum_{s=0}^{N-1} e^{isn_r\,\kappa\,sin\theta+\phi'}
\end{aligned} \tag{14}$$

$$S_N = \sum_{s=0}^{N-1} (-1)^s\ e^{s\,n_{im}\kappa\,sin\theta+\phi'} \cdot \sum_{s=0}^{N-1} e^{isn_r\,\kappa\,sin\theta+\phi'} \tag{15}$$

Let $\Psi = \kappa\, sin\theta + \phi'$

$$S_N = \sum_{s=0}^{N-1} (-1)^s\ e^{s\,n_{im}\Psi} \cdot \sum_{s=0}^{N-1} e^{isn_r\,\Psi} \tag{16}$$

References

1. Optics by Eugene Hect and A.R. Ganeshan, 4th edition, Pearson Educations.
2. Optics by M.P. Vaughan, UCC(2014)
